# Light source calibration for multispectral imaging in surgery

**DOI:** 10.1007/s11548-020-02195-y

**Published:** 2020-06-13

**Authors:** Leonardo Ayala, Silvia Seidlitz, Anant Vemuri, Sebastian J. Wirkert, Thomas Kirchner, Tim J. Adler, Christina Engels, Dogu Teber, Lena Maier-Hein

**Affiliations:** 1 Division of Computer Assisted Medical Interventions, German Cancer Research Center (DKFZ), Heidelberg, Germany; 2 HIDSS4Health – Helmholtz Information and Data Science School for Health, Karlsruhe, Heidelberg, Germany; 3 Faculty of Mathematics and Computer Science, Heidelberg University, Heidelberg, Germany; 4 Medical Faculty, Heidelberg University, Heidelberg, Germany; 5 Urologische Klinik, Städtisches Klinikum Karlsruhe, Karlsruhe, Germany

**Keywords:** Specular highlights, Dichromatic reflection model, Illuminant spectral estimation, Multispectral imaging, Perfusion imaging, Surgical data science

## Abstract

**Purpose:**

Live intra-operative functional imaging has multiple potential clinical applications, such as localization of ischemia, assessment of organ transplantation success and perfusion monitoring. Recent research has shown that live monitoring of functional tissue properties, such as tissue oxygenation and blood volume fraction, is possible using multispectral imaging in laparoscopic surgery. While the illuminant spectrum is typically kept constant in laparoscopic surgery and can thus be estimated from preoperative calibration images, a key challenge in open surgery originates from the dynamic changes of lighting conditions.

**Methods:**

The present paper addresses this challenge with a novel approach to light source calibration based on specular highlight analysis. It involves the acquisition of low-exposure time images serving as a basis for recovering the illuminant spectrum from pixels that contain a dominant specular reflectance component.

**Results:**

Comprehensive in silico and in vivo experiments with a range of different light sources demonstrate that our approach enables an accurate and robust recovery of the illuminant spectrum in the field of view of the camera, which results in reduced errors with respect to the estimation of functional tissue properties. Our approach further outperforms state-of-the-art methods proposed in the field of computer vision.

**Conclusion:**

Our results suggest that low-exposure multispectral images are well suited for light source calibration via specular highlight analysis. This work thus provides an important first step toward live functional imaging in open surgery.

## Introduction

**Fig. 1:**
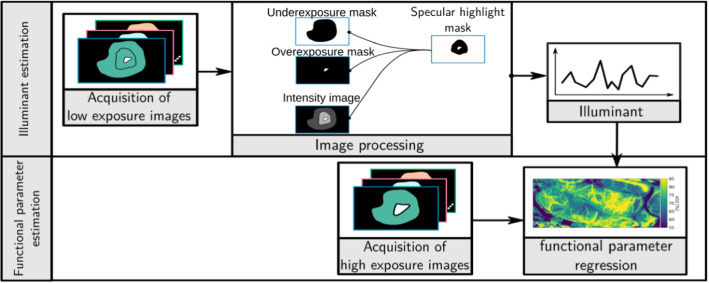
Concept overview. Based on low-exposure multispectral images, *specular highlight masks* are computed, which represent regions in the image that are not over- or underexposed and are assumed to contain a dominant specular component. A machine learning algorithm adapted to the illuminant estimate is then applied to high-exposure multispectral images to compute functional tissue parameters

The most commonly applied approach to computer-aided surgery (CAS) relies on intra-operative registration of preoperative images with the current patient anatomy. While this concept has the potential to significantly enhance surgical perception, it has one major bottleneck: It cannot account for tissue *dynamics* as the superimposed information has been extracted from “offline” images taken prior to surgery. Recent developments have shown that multispectral imaging (MSI) has the potential to overcome this drawback by enabling the live extraction of functional tissue parameters [19]. Despite the recent success of functional MSI in the field of minimally invasive surgery, a key challenge related to transferring the technique to open surgical procedures is the lack of accurate information on the illumination conditions. Due to multiple different light sources (e.g., overhead lights, ceiling lights, head torches) being present and moved during the surgical procedure, the combined illuminant spectrum at the surgical site changes drastically and dynamically. Previous approaches have addressed this issue by requiring the surgical lights to be turned off and/or by enforcing static illumination conditions (e.g., no movement of the light sources) [8, 14]. Both solutions can be seen as severe interference with the surgical workflow.

To overcome this bottleneck, we propose the first approach to live illuminant spectrum estimation in the operating room (OR). As illustrated in Fig. 1, the core idea is to capture low-exposure images, from which the illuminant spectrum in the field of view of the camera can be estimated via specular highlight analysis. The estimation of functional tissue parameters is then performed on standard high-exposure images with a machine learning method [18] adapted to the (current) illuminant estimate. The remaining part of this paper is structured as follows. As we are not aware of any prior work on automatic illuminant estimation in surgery, we review the related work on illuminant estimation outside the medical domain (Section “Related work”). Next, we present our approach to illuminant estimation (Section “Materials and methods”) along with the in silico and *ex vivo* experiments we performed to validate our approach (Section “Experiments and results”). We then conclude with a discussion of our findings in Section “Discussion”.

## Related work

Illuminant estimation refers to the estimation of the illuminant spectrum in the field of view of the camera from one or multiple images. Note that illuminant estimation is strongly related to *color constancy (CC)* methods [13], which are developed with the aim of perceiving the color of objects independently of the color of illumination. They are typically developed for computer vision applications [1] and can be classified in two main groups: *model-based methods* and *machine learning methods*.

*Model-based methods* Model-based methods use basic assumptions on the image formation process to extract the illuminant directly from (single) images. Khan *et. al.* [13] recently identified the four most widely used methods for RGB image data and described how to extend them to multispectral images. **M1: Max-RGB** is based on Land’s white patch algorithm [15], which states that there is at least one pixel in each channel of an image that produces maximum reflection of the illuminant. Combining the maximum reflection from each channel, the illuminant can be recovered. **M2: Gray-world** is based on the assumption that the average value of each channel computed over one image is achromatic and contains only information of the illuminant. Combining the average from each channel, the illuminant can be recovered. **M3: Shades-of-gray** is a generalization of **M1** and **M2**, where **M1** is equivalent to using $L^{\infty }$ normalization on each channel, while **M2** is equivalent to using $L^1$ normalization. Given a multispectral image $I \in \mathbb {R}^{N_x \times N_y \times N_s}$ of spatial dimensions $N_x \times N_y$ and number of channels $N_s$, $I_k \in \mathbb {R}^{N_x\times N_y}$ represents one image channel *k*$(k \in \{ 1,\ldots ,N_s \})$ and $(I_k)_{(i, j)} \in \mathbb {R}$ represents the intensity value at the pixel position (*i*, *j*) ($i \in \{ 1,\ldots ,N_x \}$, $j \in \{ 1,\ldots ,N_y \}$). With *p* being the order of the Minkowski norm, the estimated illuminant $L_k$ is derived as follows:

$$\begin{aligned} \left( \frac{\iint |I_k|^p dxdy}{\iint dxdy} \right) ^ {1/p} \approx \left( \frac{\sum _{(i,j)}^{N_x,N_y} |I_k|_{(i,j)}^p}{N_x \cdot N_y} \right) ^ {1/p} \propto L_k \end{aligned}$$

For **M3**, *p* is set to 6 following the suggestion of Finlayson *et. al.* [5]. **M4: Gray-edge** is based on the assumption that the average of the reflectance derivative in a scene is achromatic. This can be expressed as follows

$$\begin{aligned}&\left( \frac{\iint |I_k^{\prime \prime \sigma }|^p dxdy}{\iint dxdy} \right) ^{1/p} \approx \left( \frac{\sum _{(i,j)}^{N_x,N_y}|I_k^{\prime \prime \sigma }|^p_{(i,j)}}{N_x \cdot N_y} \right) ^{1/p} \propto L_k\\&I_k^{\prime \prime \sigma } = \sqrt{(\partial _i I_k^{\sigma })^2 + ( \partial _j I_k^{\sigma })^2} \end{aligned}$$

where $(I_k^{\prime \prime \sigma })$ represents the smoothed derivative of image channel *k* with a Gaussian filter of standard deviation $\sigma$. Following the recommendation of [5], *p* is set to 6.

Other widely used methods are based on the *dichromatic reflection model* [10] which states that the light reflected from an object can be separated into a specular and a diffuse reflection component.

$$\begin{aligned} (I_k)_{(i,j)} = {\overbrace{(S_k)_{(i,j)}L_k}^{diffuse}} + {\overbrace{c\cdot L_k}^{specular}} \end{aligned}$$

where $(I_k)_{(i,j)}$ is the intensity of image channel *k* at position (*i*, *j*), $(S_k)_{(i,j)}$ is the surface spectral reflectance, *c* is a constant and $L_k$ is the illuminant spectrum in channel *k*. In this approach, reflections from objects are projected to a two-dimensional space via principal component analysis (PCA), specular and diffuse clusters are then identified and a linear fit of the specular cluster yields the illuminant. Yet, according to our experience, the data acquired from complex surgical scenarios do not generally allow for straightforward identification of clusters in the PCA data. Moreover, the labeling of clusters (if any) is also challenging because there is generally no dominant specular component. This is in strong contrast to imaging data representing dielectric materials where the specular component is dominant and the corresponding cluster is bigger and clearly separable.

Other model-based approaches either are only applicable to RGB images [6] or rely on restrictions not fulfilled by the target images acquired in a surgical setting. For example, [12] assume that the object surface is composed of a dielectric material such as plastic or paint. [11] require materials to be uniform and relies on a statistical daylight model, which is not available for surgical scenarios. Furthermore, most of the methods in [13] assume that the average color of a scene is achromatic, which is a strong restriction for surgical scenarios.

*Machine learning methods* Among machine learning approaches to illuminant estimation, convolutional neural networks are widely used to estimate single and multiple scene illuminants [3, 4, 9]. Some of these approaches work on the whole image, yielding one illuminant estimation for the whole scene, while others work on patches, yielding one illuminant estimation for each patch. Some of them enable the estimation of multiple illuminants for each patch [16]. A challenge related to machine learning algorithms is that the training of these algorithms requires ground-truth knowledge on the illuminant, usually obtained by placing a color checker on each scene. However, the spectral mixing of different light sources can change during the procedure rendering accurate calibration of the light source based on preoperative or postoperative data infeasible.

*Summary* To our knowledge, no prior work on illuminant estimation for MSI in surgery has been proposed to date. Methods proposed outside the field of medicine typically suffer either from unrealistic model assumptions (model-based approaches) or the need to acquire labeled training data (machine learning-based approaches).

## Materials and methods

This section presents the multispectral camera and light sources (LS) used for our study (Section “Multispectral camera and light sources”), our method for LS calibration (Section “Automatic light source calibration”), hyperparameter settings for our proposed method (Section “Hyperparameter settings”) and our framework for generating in silico data (Section “Simulation framework for validation”).

### Multispectral camera and light sources

**Fig. 2:**
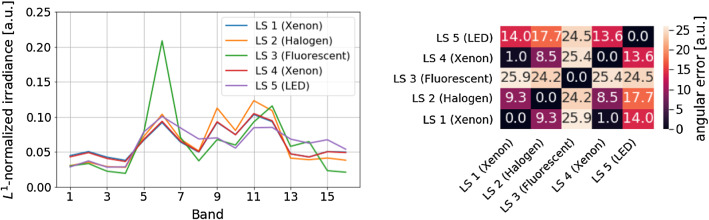
Reference light sources (LS). LS 1: xenon (D-light P 201337 20 endoscopic light source, Karl Storz GmbH, Tuttlingen, Germany); LS 2: halogen (Halopar 16 GU10 light bulb, Osram^®^, Munich, Germany); LS 3: fluorescent light (FLS 11W 2700K fluorescent lamp, Paulmann^®^, Springe Völksen, Germany); LS 4: xenon (Auto LP 5131 endoscopic light source, Richard Wolf GmbH, Knittlingen, Germany); LS 5: light-emitting diode (Endolight LED 2.2 endoscopic light source, Richard Wolf GmbH, Knittlingen, Germany). **a** Relative irradiance of LS 1-5 normalized with the $L^1$ norm. **b** Distance matrix showing the *angular error* between the different LS

We used a xiSpec MQ022HG-IM-SM4X4-VIS snapshot mosaic camera (XIMEA^®^, Münster, Germany) which records multispectral images at 16 bands in the visible range at a resolution of $512 \times 272$ pixels at video frame rate. Five different LS were used to validate our approach. The spectra of these are shown in Fig. 2 and represent some of the common illumination conditions in the OR. The reference (gold standard) illuminant spectra of all LS were obtained with an HR2000+ spectrometer (Ocean Optics^®^, Largo, USA) over a Spectralon^®^ SRT-99-050 diffuse reflectance standard (Labsphere^®^, North Sutton, USA) [2]. To quantify the difference between two LS, we consider their illuminant spectra as vectors and compute the Euclidean angle between them as proposed in [13], which we refer to as *angular error*. The angular error for the five LS used in this study ranges from 1.0$^\circ$ (LS 1 and LS 4; both xenon) to 25.9$^\circ$ (LS 1 and LS 3; xenon and fluorescent) as depicted in Fig. 2b.

### Automatic light source calibration

Our approach to automatic LS calibration is illustrated in Fig. 1 and comprises the following three main steps:

**Acquisition of calibration images** Our original idea was to recover the illuminant directly from the specular highlights of standard multispectral images (i.e., high exposure time). However, we observed that specular reflections typically saturate the detector, leading to “invalid” pixels. Other parts of the image, on the other hand, are typically substantially affected by underlying tissue properties and thus not well suited for the recovery of the illuminant. To overcome this problem, we propose the acquisition of dedicated LS calibration images, which are typically images acquired with a lower exposure time compared to that used for the multispectral images that serve as a basis for physical parameter estimation. While these (low-exposure) images are generally associated with a low signal-to-noise ratio (SNR), they are acquired in a way that “valid” specular highlight pixels (not overexposed and not underexposed) contain maximum information about the illuminant. In order to determine the optimal exposure time for these images, we performed several experiments that empirically establish a metric to determine the optimal exposure time. These experiments are detailed in Section “Experiments and results”.

**Specular highlight segmentation** Our approach to specular highlight segmentation involves removing overexposed and underexposed pixels by selecting pixels with intensities $I_{ms}$ in a specific range $I_{min}< I_{ms} < I_{max}$. The minimum intensity $I_{min}$ is set to the level of dark current for a given exposure time, determined once for each camera. The maximum intensity $I_{max}$ accounts for the nonlinearity in the camera response at high intensities and is set according to manufacturer specifications (here $I_{max}=950$). Excluding underexposed and overexposed pixels results in a set of pixel indices corresponding to “valid” pixels $\mathbb {V}$. Based on this index set, specular highlight pixels are identified as follows. Initially, the *lightness*$(I_L)_{(i,j)}$ is computed for all $(i,j) \in \mathbb {V}$ by averaging the reflectance over all bands: $(I_L)_{(i,j)} = \sum _{k=1}^{N_s} \frac{(I_k)_{(i,j)}}{N_s}$, where $N_s$ is the number of bands and $(I_k)_{(i,j)}$ is the intensity corresponding to band *k* at pixel (*i*, *j*). From the “lightness image,” a number of $N_P$*highlight pixels* with the highest values of $(I_L)_{(i,j)}$ are selected. The corresponding indices are represented by $\nu _{hl} \subseteq \mathbb {V}$. Based on an empirical analysis (see Section “Experiments and results”), we set $N_P = 100$.

**Estimation of illuminant** The illuminant is computed based on the assumption that the diffusely reflected light from the tissue can be neglected in specular highlight pixels. For each $(i,j) \in \nu _{hl}$, an estimate of the illuminant is computed by normalizing the acquired spectra $(I_k)_{(i,j)}$:

$$\begin{aligned} (L_k)_{(i,j)} = \frac{(I_k)_{(i,j)}}{||I_{(i,j)}||_1} \end{aligned}$$

where $(L_k)_{(i,j)}$ represents the estimated illuminant spectrum in band *k* from a pixel at position (*i*, *j*). The final illuminant estimation $L_k$ in band *k* is then set to the mean of all illuminant estimations from $N_p$ single pixels:

$$\begin{aligned} L_k = \frac{1}{N_P}\sum _{(i,j)}(L_k)_{(i,j)\in \nu _{hl}} \end{aligned}$$

### Hyperparameter settings

We empirically determined the appropriate values for the two hyperparameters: the exposure time $T_{exp}$ (for the calibration images) and the number of highlight pixels $N_P$ per image. We performed initial experiments using three of the five LS summarized in Fig. 2, namely LS 1-3. We refer to these LS as *validation LS*, while we refer to LS 4-5 as *test LS*. We observed that varying $N_p$ in the range of $75-200$ had a negligible influence on performance and thus set $N_p=100$. When analyzing the low-exposure images in the validation set (exposure times between 5 ms and 150 ms), we further found a goodness metric *G*, for which the angular error of illuminant estimations decreases as *G* increases, where we define *G* as:

$$\begin{aligned} {G}(T_{exp})= & {} \text {median}_{((i,j)\in \nu _{hl}(T_{exp}))}\nonumber \\&\quad \left( \frac{(I_L)_{(i,j)}(T_{exp}) -\overline{D}(T_{exp})}{\overline{D}(T_{exp})}\right) \end{aligned}$$

with $\overline{D}(T_{exp})$ corresponding to the mean lightness value obtained for dark current (lights turned off) and $\nu _{hl}(T_{exp})$ representing the indices of the highlight pixels for exposure time $T_{exp}$. Note that *G* is positive (as $\overline{D}(T_{exp})$ is small) and does not necessarily increase with exposure time due to overexposed specular highlight pixels. Based on these findings, we suggest to acquire multiple exposure images (5–150 ms, every 5 ms) and to then set $T_{exp}$, such that the corresponding (low-exposure) image has the maximum *G*. Note that we also investigated acquiring multiple images of the same $T_{exp}$ and averaging the corresponding results, but did not find an improvement with this approach.

### Simulation framework for validation

To generate in silico data for quantitative validation, we closely follow the work in [19]. In our framework, a multispectral imaging pixel is generated from a vector $\mathbf {t}_i$ of tissue properties, which are assumed to be relevant for the image formation process. Like [19], we assume a 10-valued vector comprising optical tissue properties (e.g., scattering properties and oxygenation) and properties related to the layered structure of the tissue (e.g., layer thickness). The tissue model used has 3 layers with thickness ranging from 20 to 2000 mm. Blood volume fraction on each layer is set between 0% and 30%, and blood oxygen saturation is varied between 0% and 100%. To convert a vector of tissue properties to a reflectance spectrum $\mathbf {r}_{\text {sim}}(\lambda , \mathbf {t}_i)$ (where $\lambda$ corresponds to the wavelength), the Monte Carlo method is applied. The intensity in band *k* of a pixel for a given LS and camera is then computed as

$$\begin{aligned} (I_k)_{(i,j)}(\mathbf {t}_i)= & {} \alpha _{(i,j)} \cdot w_k \, \int _{\lambda _{\text {min}}}^{\lambda _{\text {max}}}\xi _k(\lambda )\cdot \mathbf {r}_{\text {sim}}(\lambda ,\mathbf {t}_i) \,\text {d}\lambda \\&~~~ \forall k \in \{1, \ldots , N_s\} \end{aligned}$$

where (*i*, *j*) represents spatial coordinates in the image, $\alpha _{(i,j)}$ accounts for constant multiplicative changes of reflectance, $w_k$ accounts for the noise of band *k* (shot noise due to the particle nature of light, which can be approximated as multiplicative Gaussian noise in the limit of large image intensities [7]), $\xi _j(\lambda )$ represents the irradiance of the illuminant (e.g., xenon or halogen) and other components in the imaging system, such as transmittance of optical systems, and $N_s$ is the number of camera bands. By drawing samples $\mathbf {t}_i$ from the layered tissue model and generating corresponding measurements, a data set of simulated multispectral measurements with corresponding ground-truth oxygenation can be generated.

## Experiments and results

We investigated the following research questions (RQs):

- **RQ1** How accurate and robust is our approach to estimating the spectrum based on specular highlight analysis? This is addressed in Section “Accuracy of light source calibration”
- **RQ2** What is the effect of errors in the estimation of the spectrum of the LS on the accuracy of functional parameter estimation? This is addressed in Section “Effect on oxygenation estimation”
- **RQ3** How does the proposed method perform compared to state-of-the-art methods (**M1–M4**)? This is addressed in Section “Comparison to state-of-the-art methods”.

### Accuracy of light source calibration

To address **RQ1**, we gathered multispectral images of an *ex vivo* pig liver illuminated with the five LS described in Fig. 2. To determine the robustness of our approach to illuminant estimation, we acquired images corresponding to a total of eight different poses of the camera relative to each LS (at different angles and distances). Images were recorded at different exposure times (5–150 ms). To quantitatively assess the performance of our method for illuminant estimation, we applied our method to a total of $5 \times 8 = 40$ (number of LS $\times$ number of poses per LS ) images. We then computed descriptive statistics for the angle between the reference spectrum and the estimated spectrum.

**Fig. 3:**
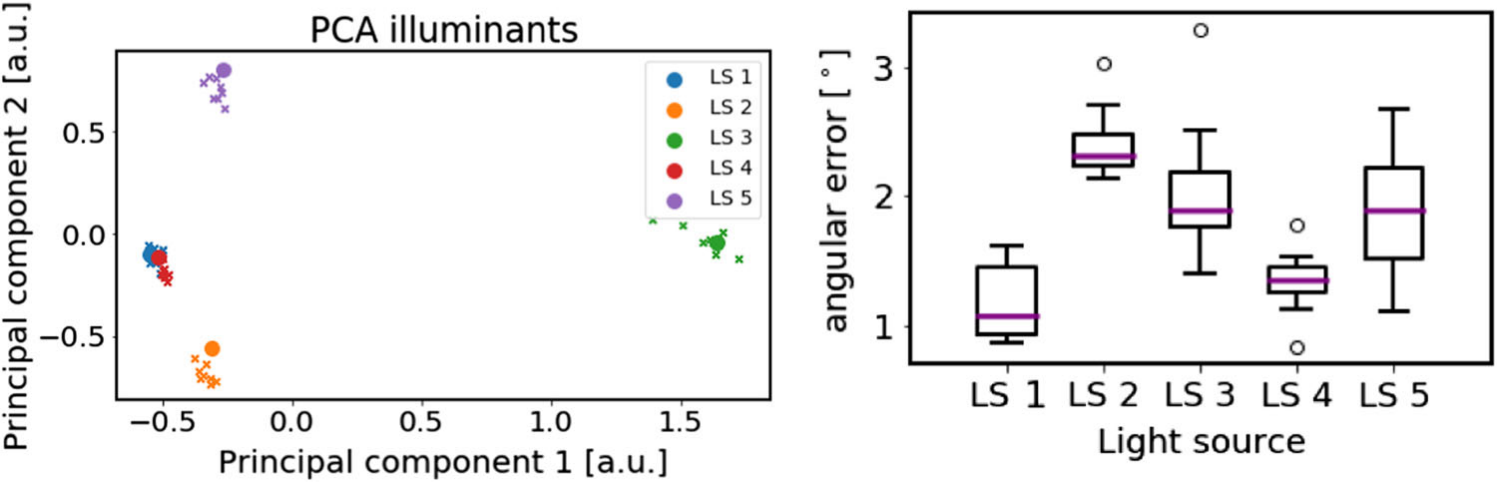
Performance of illuminant estimation. **a** Reference spectra (large circles) and corresponding estimates (crosses) for the light sources (LS) described in Fig. 2. All illuminant spectra were projected onto the first two principal components of the five reference spectra, determined with principal component analysis (PCA). Crosses of the same color represent different poses of the multispectral camera relative to the same LS. **b** Box plots of the angle between the reference spectrum and the estimated spectrum for each LS

Figure 3a shows the reference illuminant spectrum (large blobs) along with our estimations (crosses) on the first two principal components of the five reference illuminants, where symbols with the same color correspond to the same LS. The true illuminant is consistently the nearest neighbor to the estimates with the exception of LS 1 and LS 4, which are both xenon LS from different manufacturers and have an angular error of only $1^{\circ }$. The performance of our illuminant estimation method is summarized in Fig. 3b. It can be seen that the angle between our estimate and the reference is always below $3^\circ$. The performance for the *test LS* LS 4–5 is similar to those of the *validation LS* LS 1–3, which were used to tune the two hyperparameters ($T_{exp}$ and $N_p$).

**Fig. 4:**
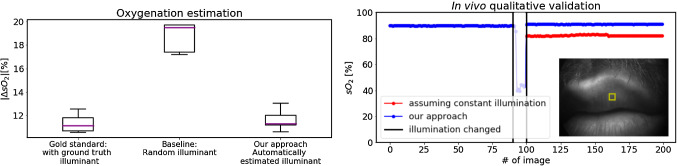
**a** Error in oxygenation estimation when (1) using the reference illuminant for training ($LS_\mathrm{train} = LS_\mathrm{test}$), (2) using a random illuminant for training ($LS_\mathrm{train} \ne LS_\mathrm{test}$) and (3) using our approach to illuminant estimation to estimate the LS ($LS_\mathrm{train} = \hat{LS}_\mathrm{test}$). **b** In vivo qualitative validation. When assuming a constant LS, the estimated blood oxygenation in an ROI on the human lips (yellow rectangle) changes when illumination conditions alter (and no longer match the training conditions). Our approach compensates for this by automatic LS calibration. The gap between index 85 and 100 represents the transition phase in which light sources were switched on and off, respectively

### Effect on oxygenation estimation

To quantify the impact of the error in illuminant estimation on the resulting oxygenation estimation error (**RQ2**), we used the simulation pipeline presented in “Simulation framework for validation” section to simulate a set of ground-truth optical properties *O* with $|O| = 15,000$, which was divided into a training data set $O_\mathrm{train}$ with $|O_\mathrm{train}| = 10,000$ and a testing data set $O_\mathrm{test}$ with $|O_\mathrm{test}| = 5,000$. $O_\mathrm{train}$ was used to generate $5+40=45$*training sets* following the approach presented in “Simulation framework for validation” section, each corresponding to one of the five ground truth LS ($LS_i \quad \forall i \in \{1, 2, 3, 4, 5\}$) or their estimates $\hat{LS}_i$ ($n=40$; one for each LS and each of the eight poses) and each comprising 10,000 tuples of tissue properties and corresponding measurements. Note that the training sets for the different illuminants correspond to the same ground-truth tissue parameters (including oxygenation, which is the parameter we wish to recover). For each training data set, we then trained a regressor for oxygenation estimation using the approach in [19]. For testing the performance of the regressors, we used $O_\mathrm{test}$ to generate a test set for each of the five reference LS, following the approach presented in Section “Simulation framework for validation” and each comprising 5000 tuples of tissue properties and corresponding measurements. We then computed descriptive statistics for the quality of oxygenation estimation: (1) using the reference illuminant for training ($LS_\mathrm{train} = LS_\mathrm{test}; n = 5$), (2) selecting randomly one of the other illuminants for training ($LS_\mathrm{train} \ne LS_\mathrm{test}; n = 20$) and (3) using our approach to illuminant estimation to estimate the LS ($LS_\mathrm{train} = \hat{LS}_\mathrm{test}; n = 40)$.

**Fig. 5:**
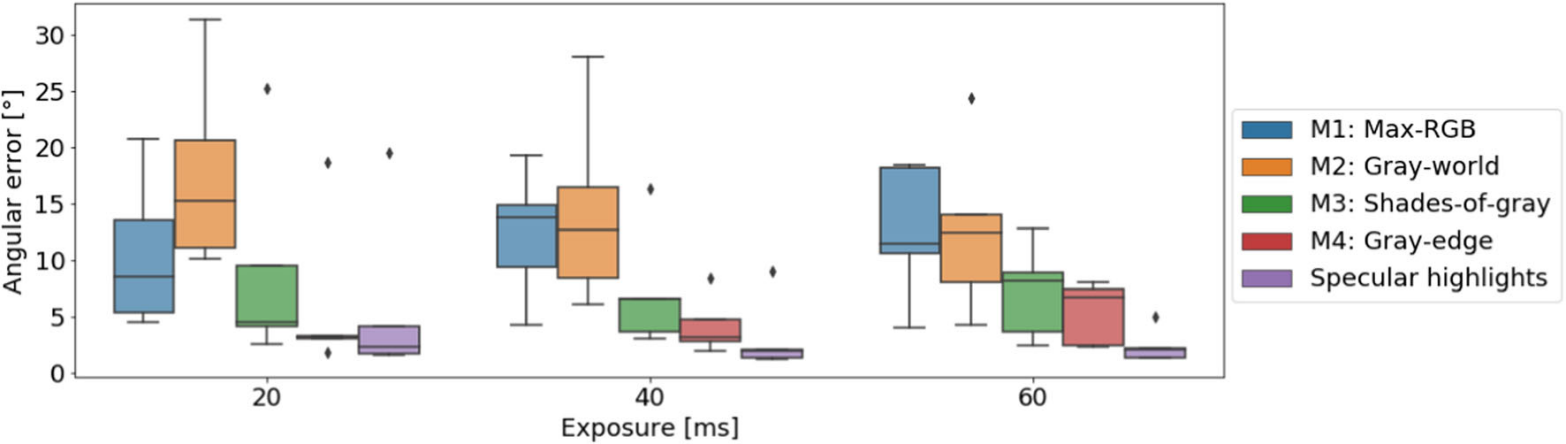
Angular error of baseline methods and our proposed method (Specular highlights) for different exposure times. Box plots of the same color correspond to the same method

For *qualitative* validation, we acquired a multispectral imaging stream from the lips of a human subject and switched the LS from LS 1 to LS 5 during recording. We applied our approach to automatic light source calibration to continuously update the regressor to one tailored to the (estimated) light source. As a baseline method, we applied a regressor trained on LS 1 (the first LS used) throughout the whole acquisition process. Qualitative analysis was performed by plotting the mean oxygenation in a region of interest (ROI).

As shown in Fig. 4a, the mean absolute error in oxygenation estimation when using the ground-truth illuminant for training ranges from 10.5pp (percentage points for LS 1) to 12.5pp (LS 3) with a mean of 11.3pp (averaged over all five LS). The mean, median and max values of the mean oxygenation error were 11.6pp, 11.3pp and 13.1pp when applying our approach ($n=40$). The results were similar for the *validation LS* (mean 11.3pp) and the *test LS* (mean 12.0pp). Compared to using an arbitrary LS (no calibration performed), we reduced the mean oxygenation error by an average of 47%. Figure 4b illustrates the benefit of our approach in vivo.

### Comparison to state-of-the-art methods

To address **RQ3**, we analyzed the state of the art in illuminant estimation and picked four related methods that fit our requirements, namely that (1) no supervised training is needed and (2) no assumption about homogeneity or composition of the surface is needed. Following the terminology introduced in Section “Related work”, we refer to these methods as (**M1–M4**). M1–M4 were applied to the recordings described in section “Accuracy of light source calibration” to perform a comparison with our approach on identical data sets.

Figure 5 shows the performance of all methods, quantified by the angular error introduced in Sect. 3, for three different exposure times (low 20 ms, normal 40 ms and high 60 ms) and averaged over all poses of all LS (**LS 1–LS 5**) ($n=8$). While our method outperformed all the competitors and yielded relatively consistent performance over the three exposure times, the performance of M1-M4 was more sensitive to the exposure time applied.

**Fig. 6:**
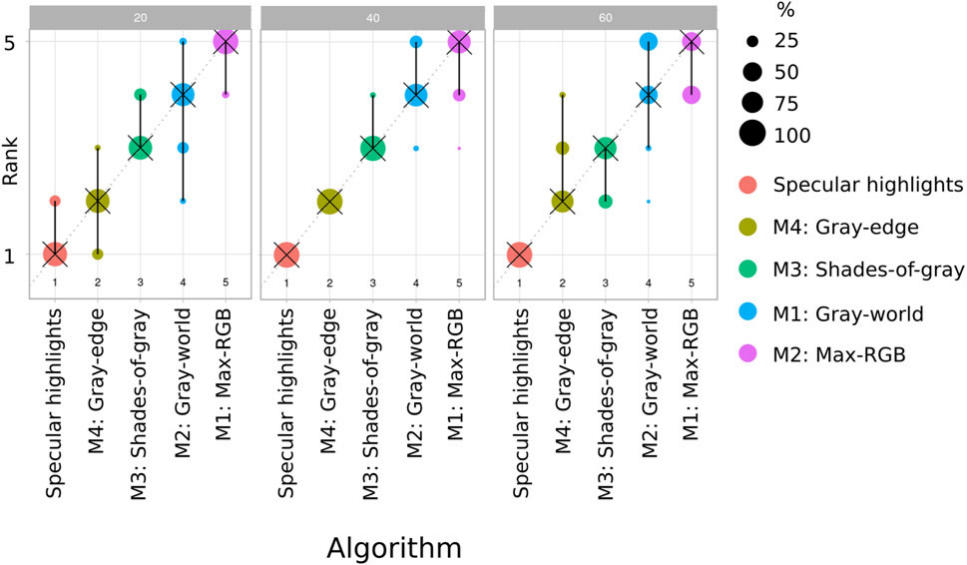
Ranking stability of different methods when applied to different exposure times. Here, the rank of a method on each data set (1 best to 5 worst) is based on the angular error. Each method is color-coded, and the area of each blob at position ($A_i, rank~j$) is proportional to the relative frequency ($A_i$) each method achieved rank *j* for 1000 bootstrap samples across the different tasks, where one task represents one exposure time. The median rank of each algorithm is indicated by a black cross and 95% bootstrap confidence intervals across bootstrap samples are indicated by black lines

A systematic robustness analysis using the *ChallengeR* tool developed by Wiesenfarth et al. [17] confirmed our hypothesis that our method is superior compared to the state-of-the-art approaches (Fig. 6).

## Discussion

To our knowledge, we are the first to propose an approach to illuminant estimation that can be applied to multispectral imaging in a surgical setting. While the machine learning algorithm for recovering tissue parameters given the LS has already been adapted from previous work [18, 19], the methodological innovation of the present paper is mainly related to the LS estimation. The guiding hypothesis that specular highlights extracted from low-exposure multispectral images can be processed to recover the illumination spectrum with high accuracy has been confirmed in our experimental analysis (**RQ1**). We further showed that the high quality of our estimations results in a high accuracy for recovering oxygenation (**RQ2**). While we optimized our hyperparameters $T_{exp}$ and $N_P$ on a subset of the LS used in our study, we did not observe a decrease in accuracy on the *test LS*. We attribute this to the fact that the estimation results were robust to changes in these parameters. A comparison with the state-of-the-art methods introduced in Sect. 2 on an identical data set showed that our method outperforms all of the competitors and is more robust to changes in the exposure time of the multispectral camera. A possible explanation for this phenomenon is that our method is not based on the entire image, but on specular highlight pixels.

While our approach pioneers automatic LS calibration and live functional imaging in open surgery, several limitations need to be overcome to fully exploit the potential of our method. First of all, we currently assume the illuminant spectrum to be homogeneous in the field of view of the camera. While initial experiments in a surgical environment suggest that this is a good approximation, we aim to extend our method such that different illuminants for different image patches can be computed. Secondly, we currently adapt the machine learning algorithm for oxygenation estimation by choosing a pre-trained regressor from a discrete set of regressors, each corresponding to a different LS. A more elegant approach would involve adapting a single regressor to dynamically changing lighting conditions. Finally, the capture of low-exposure images can be regarded as a disruption of the surgical workflow. We aim to compensate for this by implementing a method for illumination change detection, which would result in the acquisition of low-exposure images “on demand.” Given the high accuracy of our method compared to related methods along with the high processing speed (currently $\sim$ 50 ms), we believe that an occasional acquisition of low-exposure images ($\sim$ 1-2 s) is acceptable.

In conclusion, we have demonstrated that low-exposure multispectral images are well suited for recovering the illuminant via specular highlight analysis. This work thus presents an important first step toward live functional imaging in open surgery.

## References

[1] Agarwal V, Abidi B, Koschan A, Abidi MA (2006). An overview of color constancy algorithms. J Pattern Recognit Res.

[2] Antonio RK, Cong PH (2013) Imaging spectroscopy for scene analysis. chap. 3.2.2, pp 24–25

[3] Bianco S, Cusano C, Schettini R (2015) Color constancy using CNNs. In: IEEE computer society conference on computer vision and pattern recognition workshops 2015(2), pp 81–89

[4] Bianco S, Cusano C, Schettini R (2017). Single and multiple illuminant estimation using convolutional neural networks. IEEE Trans Image Process.

[5] Finlayson G, Trezzi E (2004) Shades of gray and colour constancy. In: The twelfth color imaging conference: color science and engineering systems, technologies, applications, pp 37–41

[6] Finlayson GD, Schaefer G (2001). Solving for colour constancy using a constrained dichromatic reflection model. Int J Comput Vis.

[7] Healey GE, Kondepudy R (1994). Radiometric CCD camera calibration and noise estimation. IEEE Trans Pattern Anal Mach Intell.

[8] Holmer A, Marotz J, Wahl P, Dau M, Kämmerer PW (2018). Hyperspectral imaging in perfusion and wound diagnostics - Methods and algorithms for the determination of tissue parameters. Biomedizinische Technik.

[9] Hu Y, Wang B, Lin S (2017) FC 4 : Fully convolutional color constancy with confidence-weighted pooling. In: IEEE conference on computer vision and pattern recognition, pp 330–339

[10] Imai Y, Kato Y, Kadoi H, Horiuchi T, Tominaga S (2011) Estimation of multiple illuminants based on specular highlight detection. In: International workshop on computational color imaging, pp 85–98

[11] Kaneko E, Aoki H, Tsukada M (2016) Daylight spectrum estimation from hyper-and multispectral image without area extraction of uniform materials. In: Proceedings—11th international conference on signal-image technology and internet-based systems, pp 53–60

[12] Kato Y, Horiuchi T, Tominaga S (2012) Estimation of multiple light sources from specular highlights. In: International conference on pattern recognition, pp 2083–2086

[13] Khan HA, Thomas J-B, Hardeberg JY, Laligant O (2017). Illuminant estimation in multispectral imaging. J Opt Soc Am A.

[14] Kulcke A, Holmer A, Wahl P, Siemers F, Wild T, Daeschlein G (2018). A compact hyperspectral camera for measurement of perfusion parameters in medicine. Biomedizinische Technik.

[15] Land EH (1977). The retinex theory of color vision. Sci Am.

[16] Shi W, Loy CC, Tang X (2016) Deep specialized network for illuminant estimation. In: European conference on computer vision, pp 371–387

[17] Wiesenfarth M, Reinke A, Landman BA, Cardoso MJ, Maier-Hein L, Kopp-Schneider A (2019) Methods and open-source toolkit for analyzing and visualizing challenge results. ArXiv preprint arXiv:1910.05121 10.1038/s41598-021-82017-6 PMC7841186 33504883

[18] Wirkert SJ, Kenngott H, Mayer B, Mietkowski P, Wagner M, Sauer P, Clancy NT, Elson DS, Maier-Hein L (2016). Robust near real-time estimation of physiological parameters from megapixel multispectral images with inverse Monte Carlo and random forest regression. Int J Comput Assist Radiol Surg.

[19] Wirkert SJ, Vemuri AS, Kenngott HG, Moccia S, Götz M, Mayer BFB, Maier-Hein Klaus, H, Elson, Daniel S, Maier-Hein L (2017) Physiological parameter estimation from multispectral images unleashed. In: Medical image computing and computer assisted interventions, pp 134–141

